# Mandatory Notification of Chronic Chagas Disease: Confronting the Epidemiological Silence in the State of Goiás, Brazil

**DOI:** 10.3390/tropicalmed5020092

**Published:** 2020-06-05

**Authors:** Liliane da Rocha Siriano, Andrea Marchiol, Marina Pereira Certo, Juan-Carlos Cubides, Colin Forsyth, Fabrício Augusto de Sousa

**Affiliations:** 1 State Coordination of Zoonoses, Epidemiological Surveillance Management (GVE), Health Surveillance Superintendence (SUVISA), Goiás State Health Secretary (SES), Goiânia 74093-250, Brazil; zoonoses.go.gov@gmail.com; 2 Access Project and Operational Research Platform for Chagas, Drugs for Neglected Diseases initiative (DNDi), Rio de Janeiro 20010-903, Brazil; amarchiol@dndi.org (A.M.); cforsyth@dndi.org (C.F.); 3 Brazilian Medical Unit-BRAMU, Doctors without Borders (MSF), Rio de Janeiro 20040-006, Brazil; juan.cubides@rio.msf.org

**Keywords:** Chagas disease, disease notification, public policy, neglected topical diseases, healthcare access

## Abstract

Objectives: This paper presents the results of the design and implementation process for the policy of compulsory notification of chronic Chagas disease in the Brazilian state of Goiás (Resolution No. 004/2013-GAB/SES-GO). Methods: The narrative was based on information provided by key actors that were part of the different stages of the process, built on contextual axes based on participants’ reflections about the establishment of the most accurate and coherent notification mechanisms. Results: The notification policy addressed the absence of historical data from patients in the state Chagas program, an increase in cases identified through serology, and weaknesses in vector control. Two key challenges involved human resources capacity and dissemination to public agencies and health care workers. Effective training and communication processes were key ingredients for successful implementation. Conclusions: The lack of public health measures aimed at the epidemiological surveillance of chronic Chagas cases constitutes a significant barrier for patients to access appropriate diagnosis, management and follow-up, and hampers the planning of necessary activities within health systems. The implementation of the notification policy in Goiás allows authorities to determine the real magnitude of Chagas disease in the population, so that an appropriate public health response can be mounted to meet the needs of affected people, thereby ending the epidemiological silence of Chagas disease.

## 1. Introduction

Chagas disease is classified by the World Health Organization (WHO) as part of the Neglected Tropical Diseases (NTDs) group [1]. NTDs are characterized not only by their substantial social impact in various settings throughout the world but also by gaps in epidemiological surveillance and a lack of effective diagnostic and therapeutic tools to bolster control initiatives [2]. There are relatively scarce epidemiological data available for these often-hidden diseases, making it difficult to accurately assess their burden, and impairing the ability of governments to respond with appropriate public policies.

The International Statistical Classification of Diseases and Related Health Problems (ICD) systematizes data for Chagas disease and other conditions. This classification is based on a notification system, defined as the communication to public health authorities of the existence of a disease in humans. The purpose of this communication is to understand the magnitude and epidemiological characteristics, as well as establish control measures to prevent the spread of diseases. This information also enables the planning, execution, and evaluation of publicly controlled policies. The case of Chagas disease is especially complex due to its particular biological context (with a typically unrecognizable acute phase and long asymptomatic period) and social dimensions (primarily affecting marginalized populations including migrants and the rural poor). These conditions, along with severe gaps in diagnostic coverage, make it difficult to monitor current surveillance systems.

Reporting of the acute form of Chagas disease is mandatory in Brazil, as it is for any other disease with an acute presentation with significant morbidity and mortality [3]. However, in its chronic form, notification of Chagas disease is not mandatory in most endemic countries. This could represent a barrier in surveillance systems in terms of detection, monitoring, and health interventions considering early diagnosis and treatment [4]. The occurrence of a greater socioeconomic and public health impact of the disease is documented in its chronic phase [5]. Moreover, the vast majority of cases are not detected during the acute phase. While the regional incidence in the Americas has declined to around 30,000 annually [6], there are around six million people with chronic infection, the vast majority undiagnosed. The lack of policies supporting notification of chronic cases generates a series of limitations and loss of information for surveillance systems in endemic countries, leading to an epidemiological silence or ignorance of the epidemiological situation of the disease, hampering public policy responses for the benefit of affected populations.

Incomplete and non-systematized information leads to the impossibility of evaluating potential risk scenarios, with regional implications. There are several barriers in this regard, ranging from the problems of endemic regions to the newly emerging urban and cross-border contexts, with a high flow of migrant populations. Other difficulties are found in the definition of specific targets for control programs under national and international commitments and in the exchange of information for analysis and response to new and emerging epidemiological contexts (such as oral transmission and rural-urban and transnational migration). Currently, epidemiological data provided by countries originate mainly from blood blanks, which since the 1980s have complied with specific legislation for universal screening. Other sources of information include community testing activities, serological surveys, certification initiatives for the control of vector transmission of infection and isolated interventions of academic interest [6]. However, these sources provide a largely fragmented picture of the epidemiological burden of the disease.

Brazil has made substantial progress in terms of controlling vector transmission in the last 25 years, reaching the interruption of intra-household vector transmission by *Triatoma infestans* in 2006 [7], although the state of Goiás received this certification in March 2000 [8]. Despite significant efforts to achieve these objectives, Chagas disease remains a significant public health problem in Goiás. Moraes et al. found that 14.8% of all deaths reported in the country due to Chagas disease between 2006 and 2011 were in Goiás, with the state’s mortality rate being five times higher in comparison to the rest of the Brazilian territory [9]. There was another study on the detection of communicable diseases in pregnant women in the state of Goiás. It revealed that between 2003 and 2009, 1768 seropositive people were seropositive for *Trypanosoma cruzi* infection, representing 0.5% seroprevalence [10].

Since 2013, Goiás has implemented a mandatory public notification policy at the state level for cases of chronic Chagas disease, which has served as a key contributor in the epidemiological surveillance system, improving awareness of Chagas disease. This policy undoubtedly contributed to Brazil’s decision in early 2020 to make Chagas disease reportable at the national level and provides an example that can be replicated in other areas with a similar epidemiological profile. The objective of this paper is to present the results of the design and implementation process for the policy of compulsory notification of chronic Chagas disease in the Brazilian state of Goiás.

## 2. Methods and Materials

A questionnaire was designed containing a total of 30 questions to obtain relevant information to document the process of creating and implementing a notification policy in Goiás for chronic Chagas cases. The questions were classified into main information groups based on the stages of the public policy formulation cycle model [11].

**Figure 1:**
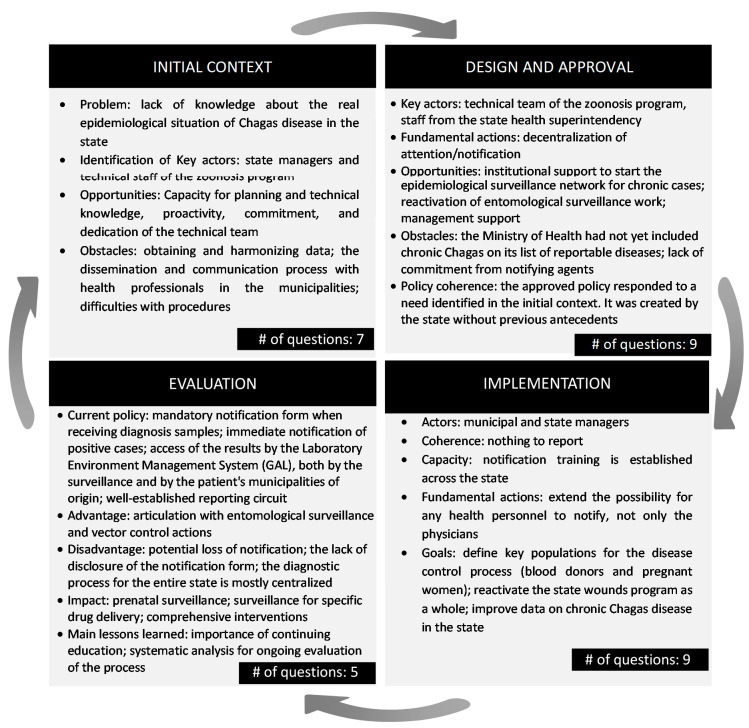
Main information axes for interviews on notification of chronic Chagas disease in Goiás.

Four main axes were chosen for data collection: the initial context, to establish how the need/problem was identified; policy design and approval, aiming to identify key actors and actions in the process; implementation, describing resources and capabilities; and evaluation of the current situation in the progress of the policy. Figure 1 illustrates the elements analyzed in each information axis. As depicted in Figure 1, the formulation of public policies is a dynamic, cyclical process.

A total of seven key employees agreed to participate in the collection of strategic information through the self-administered questionnaire, which was administered during November and December of 2018. Participants were involved in the various operational and management activities of the design and implementation processes of the chronic Chagas case notification policy in Goiás. They had different professional profiles, including two in Biomedicine, one in Biology, one in Nursing, one in Pharmacy and two from Veterinary Medicine. Most of the questions were open-ended to facilitate the inclusion of details considered pertinent/relevant by the participant.

## 3. Results

### 3.1. Initial Context

Six female and one male respondent answered the questionnaire. Respondents spent a time range of 2–8 years in their positions, which were technical (n = 3), managerial (n = 3), or administrative (n = 1). Most interviewees affirmed the notification improved Chagas disease patient access to healthcare completely (n = 5) or at least partially (n = 1); one participant did not answer. Six out of seven felt that improved data and surveillance were necessary to give visibility to the disease. When asked if the notification resolution responded to the needs it was created to address, three completely agreed, three partially agreed, and one did not respond. However, six of seven also affirmed the policy had only been partly implemented as intended.

According to the interviews, the initiative to create the policy for Chronic Chagas Compulsory Notification was consolidated in 2012, motivated mainly by the absence of historical data from patients in the Chagas state program, the increase in cases identified through serology, and the weakening of vector control programs. It was necessary to give visibility to people affected by the disease to define mechanisms for medical care and monitoring and to establish a specialized care network that adequately responded to patients’ needs. This entailed having accurate, up-to-date data available. Along with the compulsory notification of chronic patients, a set of actions was proposed to strengthen the state Chagas program.

At the time, the expectation of notification by the municipalities in Goiás was as hopeful as it was challenging. There was a weakened, overloaded notification system for compulsory diseases, characterized by few human resources and high turnover. However, the intention of the specialized team that managed the initiative was to face the need for official data, as well as to raise awareness among municipal teams. This was despite assuming that it would probably be a gradual and slow process and that there was limited recognition of the importance of reporting chronic cases by state and local authorities and managers within the health system. The work carried out to launch the policy creation initiative at the state level took approximately one year, between April 2012 and March 2013.

### 3.2. Design and Approval

To implement the compulsory notification of chronic Chagas cases, it became necessary to justify the relevance of the information and the impact it would have on epidemiological surveillance at the state level.

The technicians of the area, through internal discussions and with partners from similar areas, were in charge of identifying specific needs through a critical review of the conditions of the state Chagas program. Simultaneously, the team presented potential action items and justifications both to the management of the area and to the superintendence of health surveillance, including the formation of a state care network for patients with Chagas disease, the need to resume work in entomological surveillance in areas in ecosystems with a probability of vector transmission resurgence, and the importance of data to estimate the number of prescriptions and address the inadequate release of medications.

This information gathering instigated a process of discussion and awareness about the need to compulsorily notify cases of chronic Chagas disease, which reached the level of a joint state and municipal health committee. The initiative was also disseminated among the health secretaries of the State of Goiás for their subsequent approval. The process ended with the drafting of the resolution for publication in April 2013.

When assessing whether the public policy resolution would be in accordance with the identified needs, the participants affirmed that it enabled the development of prevention and care actions in relation to the disease as planned, even though there were some initial difficulties in rolling out the new policy. Spreading knowledge of a new policy takes time and requires the adaptation of the professionals involved. Ideally, it would be gradually incorporated into service routines. As part of the dissemination process, the Coordinator of Zoonoses distributed technical note No. 05/2013 to the regional health departments of the state and, consequently, to the municipalities, with guidance on Chagas disease in Goiás, clinical and laboratory diagnosis, control, treatment and compulsory notification in accordance with the approved resolution.

**Figure 2:**
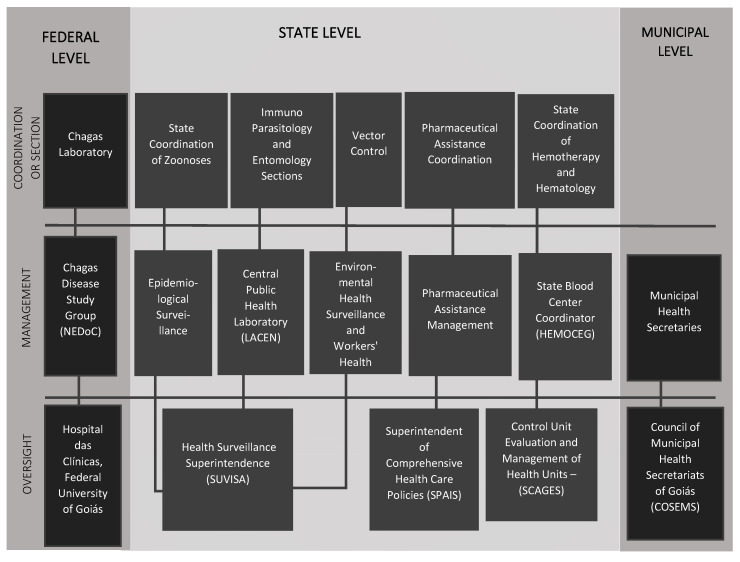
Main actors involved in the initiative to create the notification policy for chronic Chagas disease.

The initiative to create a public notification policy involved several actors at different levels, as described in Figure 2.

### 3.3. Implementation

The resolution approved by the State Department of Health went into effect on 6 May 2013, following the dissemination of the policy to regional health departments and municipalities. Implementation was carried out through the capacity building of professionals in the region and the technical support provided by the team responsible for the disease.

According to the experience of participants, there are ongoing limitations in the capacity to implement the policy in different areas. Human resources are considered the most critical point in the process due to the high turnover of health professionals, limited knowledge about the disease, and the lack of commitment from teams. However, all 18 state regional units, and their regional managers, and all 48 blood banks in the state (both private and public) were trained for notification in 2013. The state also promotes systematic training for Chagas disease, which includes training on the notification process. From 2017–2019, 960 health professionals, including doctors, nurses, and laboratory personnel, received this training in nine of the state’s 18 regional units.

Another challenge in implementing the policy was obtaining support from managers and state agencies for monitoring of the notifications. Engagement of managers achieved in implementing the compulsory notification of chronic cases in the state was motivated by the mandatory nature described in the resolution. It was also motivated by increases in media dissemination of local cases and the alerts presented by technicians of the zoonosis team.

The goals outlined as a result of the implementation of the notification were framed in improving the access of patients with chronic Chagas to the public health system. Therefore, it was possible to establish parameters for the decentralization of care and monitoring of patients; train medical teams in diagnosis and treatment; consolidate the state network to deal with complications associated with the disease, and resume the vector control programs and entomological research. The state’s annual health plan supports the financial sustainability of the program. The state has provided a budget for all activities related to notification since its implementation in 2013.

### 3.4. Evaluation

According to the opinions of the participants, the current notification of chronic cases of Chagas in the state is occurring systematically and has been quite consistent since 2014. The case investigation form has become a commonly used method for initiating laboratory diagnosis. There was a substantial increase in the reporting of chronic cases during the first 2 years of compulsory notification: from only 7 cases annually in 2013 (prior to full implementation), to 162 in the first year of the policy (2014), to 880 in 2015. Since then the reported cases have continued to increase by roughly 5%–10% each year, averaging 936 annually from 2015–2019.

**Figure 3:**
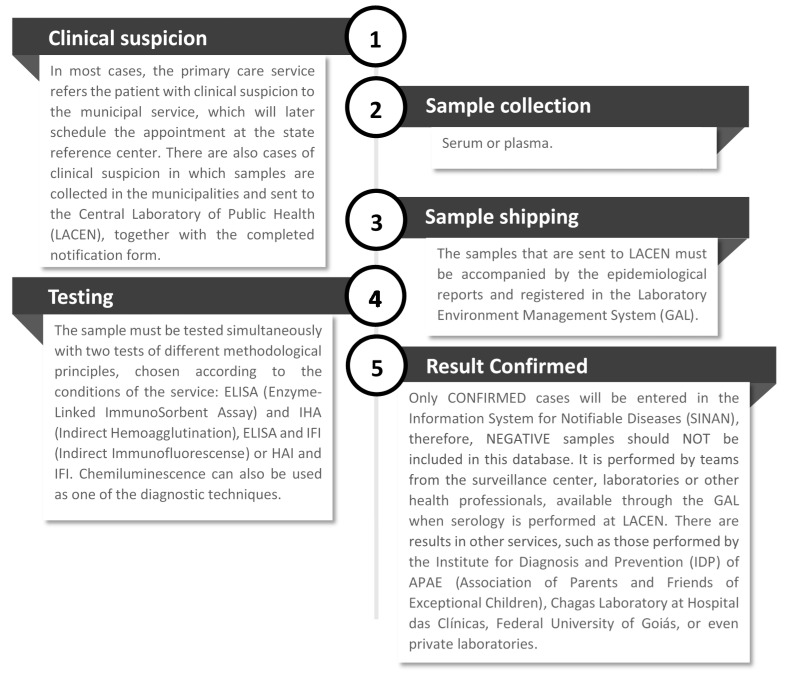
The current process of case notification of chronic Chagas disease in Goiás.

However, it is important to improve communication processes with the municipalities and continuously train medical teams so that the entire notification system is effective and efficient. The steps of the current notification process described by the interviewees are shown in Figure 3.

Only confirmed cases are entered into the SINAN (*Sistema de Informação de Agravos de Notificação*, or Information System for Notifiable Diseases). Therefore, negative samples are not included in this database. The notification is made by the core surveillance teams, by the laboratories or other health professionals, available through the Laboratory Environment Manager when the serology is performed at the Dr. Giovanni Cysneiros State Laboratory of Public Health. There are results in other services, such as those carried out by the Institute of Diagnosis and Prevention (IDP) of the Association of Parents and Friends of Special People (APAE, *Associação de Pais e Amigos dos Excepcionais*), the Chagas Laboratory at the Hospital of the Federal University of Goiás or even private laboratories.

## 4. Discussion

The lack of specific, complete and integrated information on the total number of people affected by Chagas disease frustrates the definition of public health response strategies ensuring adequate care for this population. For this reason, the creation and implementation of a public policy for notification of chronic cases in the state of Goiás is a strategic method for addressing the epidemiological silence of a highly neglected disease. Regionally, most epidemiological surveillance schemes for Chagas are limited to entomological surveillance, with or without seroepidemiological support [12,13]. In Goiás, the lack of information and activities to improve the care of people with Chagas led to the development of a specific public policy of notification. The obligation to report chronic cases thereby addresses a historical problem in the surveillance system of Goiás.

Public policies are the product of the government, but their construction is the result of a complex social interaction with the participation of various actors [14]. The stages in the design of a policy cycle correspond to a sequence of elements in the political-administrative process. They can be researched concerning its actors, their relationships, their resources, their political and social networks and their practices, typically found in each phase [15]. The main actors involved in the initiative identified several critical points that defined the initial context of the proposal, justifying the need for intervention. The cycle of neglect is fed by low visibility of people affected in the country, and limited knowledge by professionals and authorities about the disease. This culminates in the failure to report the disease and the lack of interest in preventive or control actions from some professionals and priority technical areas. The identification of chronic Chagas cases in public and private services leads to obtaining a more accurate snapshot of the population needing care, facilitating the implementation of services adapted to the needs of patients and appropriate, timely allocation of resources.

The guidelines for compulsory notification of diseases or conditions in the national surveillance system are usually generated and established by the Ministry of Health. However, states and municipalities are allowed to include other diseases or conditions, according to the local epidemiological reality. Goiás was the first state in Brazil to establish the obligation to report chronic Chagas disease. This important state challenge was supported by federal guidelines enabling the establishment of procedures for preparing a resolution with the support of state managers.

**Figure 4:**
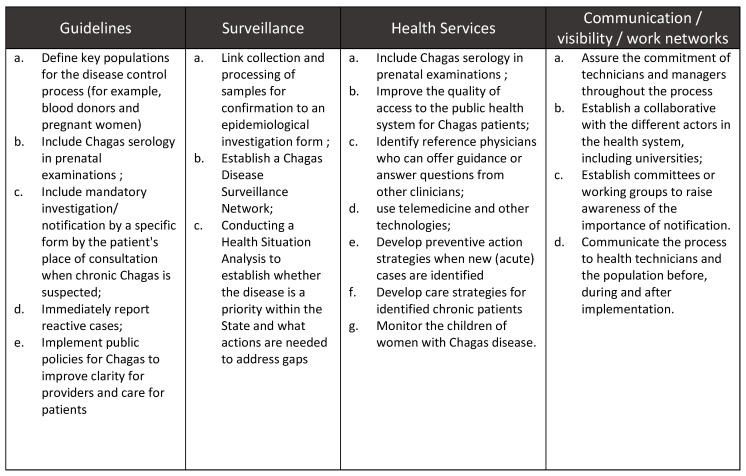
Recommendations and lessons learned from the implementation of notification of chronic Chagas disease in Goiás.

Notification of chronic Chagas disease has gained significant traction in Brazil following the adoption of the policy in Goiás. A resolution supporting notification SES/MG No. 6532 was enacted in the state of Minas Gerais in December 2018. In February 2020, an ordinance of the Brazilian Ministry of Health called for notification of chronic cases of Chagas disease throughout the national territory [16], which was updated in May 2020 [17]. This represents a significant opportunity to increase access to testing and treatment for over one million people [6] living with the disease in Brazil. The Goiás experience could serve as a guide for other states interested in strengthening local systems. Key lessons learned from the implementation of the notification of chronic Chagas disease in Goiás are shown in Figure 4.

According to local experience, the two biggest obstacles to implementation faced by the parties involved in the process were the lack of human resources and dissemination of the new guideline. Despite the recognized institutional capacity, technical knowledge, proactivity, commitment and dedication of the state technical team, these aspects continue to be significant challenges due to the high turnover of professionals at the municipal level. Clear articulation with partners from similar areas and the commitment of the technical Chagas program team have been essential to deal adequately with these implementation difficulties. Another permanent challenge for Goiás is assuring the quality of the data collected and its systematic analysis, as well as the ability to answer the training needs of health professionals addressing the mandatory notification of Chagas disease.

A vital contribution to the design of the guideline was the definition of key populations. The inclusion of blood donors and pregnant women as priority surveillance populations for the disease control process contributed to the strengthening of the system.

The notification policy has a substantial impact on the visibility of people affected by Chagas disease and in the planning and execution of public health responses. Having consolidated information that describes the real distribution of the disease in the population, as well as the factors that determine the condition of neglect, allows development of tools to achieve better prevention and control actions, reactivating the Chagas state program as a whole [18].

Chagas disease is also reportable in six states in the United States. Although the main goal of this is to identify sources of local vector transmission, these states typically include reporting of chronic cases as well. As in the case of Goiás, compulsory notification has served to strengthen awareness among health professionals, although rates of diagnosed and treated patients remain very low [19]. This underscores the fact that reporting of Chagas and other neglected diseases needs to occur in conjunction with a variety of other complementary public health actions, such as capacity building, fortifying diagnostic capabilities, and providing information to at-risk communities.

The HIV/AIDS epidemic is another important example where a notification process played a key role. Initially, infection by the virus was identified exclusively in the clinical stage of the disease as acquired immunodeficiency syndrome (AIDS). The progress achieved in diagnosis and early treatment of the infection demonstrated the opportunity and importance of surveillance for cases of HIV infection, not being limited only to AIDS cases. Thus, several factors strengthened the increase in the number of people diagnosed, such as greater access to antiretroviral therapies (ART), the importance of starting treatment at an early stage, and the implementation of mother-to-child transmission prevention programs. In this context, the surveillance of HIV infection cases and the need for their notification became increasingly relevant [11].

In the effort to make Chagas disease more visible, regional and global initiatives are discussing and urging compulsory notification in the chronic phase, including the International Federation of Associations of People Affected by Chagas Disease (FINDECHAGAS) [20], and the Chagas Disease Clinical Research Platform [21]. Notification is a gateway to a surveillance system that should guarantee an opportunity to timely medical care and, ideally, social and mental health support for affected people [22]. It also reinforces and reactivates entomological surveillance and vector control activities.

Notification of chronic cases is an essential tool which, in conjunction with capacity building of healthcare personnel, availability of diagnosis and treatment in facilities accessible to affected people, implementation of simplified diagnostic processes, and development of safer, more efficacious treatments, can usher in the end to the neglect of Chagas disease.
